# Effects of Polyphenol-Rich Foods on Chronic Diseases

**DOI:** 10.3390/nu15194134

**Published:** 2023-09-25

**Authors:** Luis Goya, Sonia de Pascual-Teresa

**Affiliations:** Departamento de Metabolismo y Nutrición, Instituto de Ciencia y Tecnología de Alimentos Nutrición (ICTAN-CSIC), C/José Antonio Novais, 10, 28040 Madrid, Spain; luisgoya@ictan.csic.es

Ever since the French paradox raised the research interest pertaining to the high potential of certain phytochemicals—until then regarded as anti-nutrients—as positive bioactive compounds for health, research on the biological and molecular effects of polyphenols has subsequently been continuously increasing. Epidemiological studies, clinical trials and interventions, animal experimentation and cell culture models have more recently benefited from new research tools that have significantly increased information and led to vast possibilities for future approaches. All these new data are necessary in order to establish nutritional claims for these compounds that might be validated by international regulating organizations and help set new healthier dietary patterns for the population as well as produce more functional foods and nutraceuticals. The present Special Issue of the journal *Nutrients* contains 16 of such new studies and reviews, which mostly report that the beneficial effects of phytochemicals validate their inclusion in a healthy human diet. A brief summary of each of the studies is offered below.

In their study on the influence of the APOE genotype, docosahexaenoic acid (DHA) and flavanol intervention on brain DHA and the lipidomics profile in aged transgenic mice, Martinsen and coworkers [1] establish a correlation between dietary flavanol intake and brain omega-3 polyunsaturated fatty acid (n3-PUFA) levels in humanized apolipoprotein E3 (APOE3) and APOE4 targeted replacement transgenic mouse models. Although only a modest—and interestingly limited to APOE3—effect on brain DHA values is observed after flavanol administration, the study by the University of East Anglia group points out the promising connection between the dietary intake of flavanols, lipid profile and brain availability of n3-PUFA [1].

Jeong-Yeon On and colleagues [2], in their study concerning the effects of fermented *Artemisia annua* L. and *Salicornia herbacea* L. on the inhibition of obesity in vitro in 3T3-L1 adipocytes and in C57BL/6 mice fed a high-fat diet, report the anti-obesity effects of the metabolites of both plant extracts after fermentation. In addition, the authors describe the inhibitory effect on adipocyte differentiation and fat accumulation. This study by the Korean group concluded that biotransformation in vitro displayed significant metabolite changes that increased the anti-obesity effects of plant extracts in mice [2].

Similarly related to the effect of food fermentation on its biological activity, Lüersen and colleagues [3], in their German–Japanese collaborative research on the anti-diabetic properties of a soy extract rich in hydroxylated isoflavones in vitro and in *Drosophila melanogaster* in vivo, show that fermentation with *Aspergillus evoked* produced an enrichment of hydroxy-isoflavones that is accompanied by an enhanced free radical scavenging activity. Both pre- and post-fermented extracts significantly showed anti-diabetic properties on oxidative stress and inflammatory markers, and the supplementation of a high-starch diet with post-fermented hydroxyl-isoflavones-rich extract decreased the triacylglyceride levels in female *D. melanogaster*, confirming its anti-diabetic properties in an in vivo model [3].

In their observational study on the dietary sources of anthocyanins and their association with consumption biomarkers and cardiometabolic risk factors, Mostafa and colleagues [4] reveal the associations between dietary intake, microbial metabolism and the cardiometabolic health benefits of anthocyanins. A targeted metabolomic analysis of a subsample of the Diet, Cancer, and Health-Next Generations (DCH-NG MAX) study with 1351 samples from 624 participants reveals two metabolites, salsolinol sulfate and 4-methylcatechol sulfate, associated with the intake of anthocyanins from berries, and inversely related to visceral adipose tissue. The group from the University of Barcelona confirms the strong dependence of plasma metabolome biomarkers of dietary anthocyanins on the dietary source in order to correlate dietary intake with cardiometabolic health benefits [4].

On the other hand, Navarro-Masip and coworkers [5] reveal changes in body weight gain and lipolysis in adipose tissue in their research on the modulation by the grape-seed proanthocyanidins of adipose tissue adaptation to obesity in rats submitted to different photoperiods and fed a cafeteria diet for five weeks. Supplementation with grape-seed proanthocyanidins for four weeks prevented excessive body weight gain under a long photoperiod, which could be explained by an increased lipolysis in the adipose tissue. The authors conclude that the impact on obesity in the adipose tissue of flavonoids is photoperiod dependent [5].

In two intervention studies—one acute and one three weeks long—on the effectiveness of a combination of green tea catechins and coffee chlorogenic acid on postprandial glycemic responses in healthy men, Yanagimoto and colleagues [6,7] report that a combined ingestion of the two phenolic compounds significantly altered the incretin response and reduced glucose and insulin levels, suggesting an effective minimum dose of 540 mg of green tea catechins and 150 mg of coffee chlorogenic acid [6]. In addition, the randomized, double-blinded, placebo-controlled crossover trial shows that the consumption of the combined phenolics enhanced insulin sensitivity and increased postprandial GLP-1 as compared to the placebo group [7].

In their research on the modulatory effect of chlorogenic acid and coffee extracts on Wnt/β-catenin pathway in colorectal cancer cells, Villota and coworkers [8] report that polyphenol-rich coffee extracts and chlorogenic acid regulate the Wnt pathway on colorectal cancer SW480 cells, with a reduction in the transcriptional activity of β-catenin. The Colombian group concludes that the results establish a starting point for the discovery of a mechanism of action of chlorogenic acid on Wnt pathway and confirm the anti-colorectal cancer potential of polyphenols present in coffee [8].

In their report on the chemical characterization, antioxidant capacity and anti-oxidative stress potential of the South American medicinal plant *Desmodium tortuosum*, Rodríguez and coworkers [9] report the relevant amount of antioxidant phytochemicals in the plant and their antioxidant capacity in vitro. Furthermore, the study shows the cytoprotective capacity of *Desmodium tortuosum* extract both in endothelial and neuronal-like cell lines subjected to oxidative stress. The Peruvian–Spanish team suggests that this chemoprotective effect must be due to the high content of phenolic compounds such as phenolic acids, flavonoids, carotenoids and other antioxidant compounds, and confirm the medicinal use of the plant [9].

In their study on the effect of hesperidin supplementation on rat mucosal immunity after an intensive chronic training and an exhausting exercise, Ruiz-Iglesias and colleagues [10] focused on fecal microbiota and composition and the function of mesenteric lymph node lymphocytes and mucosal immunoglobulin A. The authors conclude that, although hesperidin supplementation did not prevent exercise-induced changes in the distribution and function of lymphocytes, it was able to enhance immunoglobulin A synthesis in the intestinal compartment. This effect could be important in enhancing the immune intestinal barrier in this stressful situation [10].

In a metabolomics approach on the protective role of cocoa in Zucker Diabetic Rats via 1H-NMR-based approach, Fernández-Millán and colleagues [11] identified 14 differential urinary metabolites in Zucker diabetic fatty rats fed with a 10% cocoa-rich diet for 10 weeks. A correlation analysis of pathways indicated major associations between some of the urine metabolites (mainly valine, leucine and isoleucine) and body weight, glycaemia, insulin sensitivity and glycated hemoglobin levels. The authors conclude that an untargeted metabolomics approach provides a clear metabolic fingerprint associated with chronic cocoa intake that can be used as a marker for the improvement of glucose homeostasis in a diabetic context [11].

In a randomized, blinded, cross-over, controlled clinical trial carried out in normocholesterolemic and hypercholesterolemic subjects on the effect of olive pomace oil (OPO) on cardiovascular health and associated pathologies, González-Rámila and colleagues [12] report a lack of effect on any of the markers related to lipid profile, blood pressure and endothelial function in both groups. However, a significant decrease in visceral fat in both groups of subjects was observed after OPO intake, accompanied by an increment of leptin only in the hypercholesterolemic group. The authors conclude that reducing visceral fat after prolonged OPO intake might contribute to improving cardiometabolic status, with a potentially positive effect on the vascular tone [12].

In another intervention trial from the same research group, Seguido and his colleagues [13] report that the sustained consumption of a decaffeinated green coffee nutraceutical (300 mg hydroxycinnamates twice daily for two months) has limited effects on phenolic metabolism and bioavailability in overweight/obese subjects. The study of plasma and urinary pharmacokinetics, and the fecal excretion of phenolic metabolites via LC-MS-QToF, showed a significant increase in reduced forms of caffeic, ferulic and coumaric acids, or 3-(3′-hydroxypenyl)propanoic, and 3,4-dihydroxybenzoic acids in feces, and a decrease in coumaroylquinic and dihydrocoumaroylquinic acids in urine. The authors conclude that the nutraceutical product shows a small overall effect on the bioavailability of polyphenols [13].

In a comprehensive overview of in vitro and in vivo animal and human trials of the anti-diabetic potential of fruits of the rosaceae Maleae tribe, covering 131 articles published this century, Rutkowska and Olszewska [14] review the anti-diabetic effects and potential mechanisms of the action of fruits from 46 species. The first part of this review focuses on the effects on tissue-specific glucose transport and the expression or activity of proteins in the insulin signaling pathway; whereas the second part covers the phytocompounds responsible for the activity of particular fruits—primarily polyphenols (e.g., flavonols, dihydrochalcones, proanthocyanidins, anthocyanins, phenolic acids), but also polysaccharides, triterpenes and their additive and synergistic effects. The authors conclude that fruits from the Maleae tribe seem promising as functional foods for diabetes management [14].

In another overview on the polyphenol avenanthramide, a group of polyphenolic compounds found abundantly in oat (*Avena sativa* Linn.), as regulators of PI3K signaling in the management of neurodegenerative diseases, Wankhede and colleagues [15] report that these compounds have been shown to modulate PI3K/AKT signaling. Since the dysregulation of PI3K signaling has been implicated in the pathogenesis of various neurodegenerative diseases such as Alzheimer’s and Parkinson’s, the recovery of regular activity might lead to increased neuronal survival, reduced oxidative stress, and improved cognitive function. The authors conclude that Avenanthramides have emerged as promising candidates for neuroprotection due to their immense antioxidant, anti-inflammatory and anti-apoptotic properties [15].

Finally, in a review proposing the regular inclusion of pistachio in our diet, Mateos and coworkers [16] report the healthy nutritional profile of pistachio that, together with its rich composition in phytochemicals, such as tocopherols, carotenoids and, importantly, phenolic compounds, make these seeds a powerful food ingredient to explore its involvement in the prevention of prevalent pathologies. The review gathers recent data regarding the most beneficial effects of pistachio on lipid and glucose homeostasis, endothelial function, oxidative stress and inflammation that essentially result in a protective/preventive effect on the onset of pathological conditions, such as obesity, type 2 diabetes, cardiovascular disease and cancer [16].
